# Costs of Potential Medication Wastage Due to Switching Treatment Among People With Multiple Sclerosis

**DOI:** 10.36469/001c.123336

**Published:** 2024-10-28

**Authors:** Darin T. Okuda, Achal Patel, Robert Schuldt, Ibraheem Abioye, Nicole G. Bonine

**Affiliations:** 1 Department of Neurology The University of Texas Southwestern Medical Center, Dallas, Texas, USA; 2 Peter O’Donnell Jr. Brain Institute The University of Texas Southwestern Medical Center, Dallas, Texas, USA; 3 Genentech, Inc., South San Francisco, California, USA

**Keywords:** multiple sclerosis, disease-modifying therapies, site of service, cost, medication wastage

## Abstract

**Background:** Unused medications negatively impact healthcare resource utilization and environmental safety, contribute substantially to annual healthcare expenditures, and may ultimately affect patient health outcomes. People with multiple sclerosis (PwMS) commonly switch disease-modifying therapies (DMTs), leading to medication wastage and substantial costs for insurers and patients.

**Objectives:** To estimate the cost associated with potential medication wastage (PMW) in a subcohort of PwMS receiving oral or self-injectable US Food and Drug Administration–approved DMTs who switched DMTs in a calendar year in the United States.

**Methods:** This retrospective cohort study included adults with MS and used PharMetrics® Plus claims data from 2017 to 2021. PwMS were required to have 12 months of continuous eligibility for the entire year and a claim for at least 2 unique DMTs during the same calendar year. The PMW cohort was defined as those who had an aggregate overlap in days’ supply across DMT switches within the year; those in the non-PMW cohort did not. The cost of PMW for insurers and PwMS due to overlap was calculated only at the point of switch to the new DMT and defined as the cost of the remaining days’ supply of the prior DMT.

**Results:** The number of PwMS meeting the inclusion criteria was 1762 in 2017, 1947 in 2018, 1679 in 2019, 1461 in 2020, and 1782 in 2021. Approximately 95% of PwMS switched DMTs once within single calendar years, and 25% (n = 381-464) contributed to PMW. For those who had overlapping DMT supply, it was estimated that 34% to 38% of the DMT being switched from was potentially wasted. The total cost of PMW paid by the insurer and PwMS ranged from 1 200 866to1 489 859. While most of the total cost (1 172 140−1 450 328) was paid by the insurer, PwMS still owed substantial amounts (28 726−74 578). Across all PwMS, the per person per year cost ranged from 716to846. The estimated wastage and associated costs were consistent across all study years.

**Conclusions:** DMT switching is common among PwMS, resulting in PMW and high costs to patients and insurers.

## INTRODUCTION

In the US, approximately 1 million people have multiple sclerosis (MS), with 15 000 to 18 000 new diagnoses annually.^1^ Initiation of a disease-modifying therapy (DMT) soon after MS diagnosis is associated with favorable clinical outcomes and lower direct and indirect medical costs.^2^ Over 20 US Food and Drug Administration–approved DMTs are available, including oral or self-injectable medications that can be administered at home, or intravenous (IV) medications infused in healthcare settings.^3^ People with MS (PwMS) who adhere to DMTs have less frequent and severe relapses, fewer emergency department visits and hospitalizations, fewer neuropsychological issues, lower costs, and increased quality of life.^4^ However, DMT switches are common and can result from multiple factors, including breakthrough disease, lack of adherence, and intolerable adverse events.^3^

MS has an estimated economic burden of $2.5 billion in the US, with individual lifetime costs of over $4 million.^5^ Notably, costs for neurological medications have increased exponentially in recent years and are on a trajectory to become even more expensive.^6,7^ DMTs are the largest medical expenditure for PwMS, accounting for more than half of all-cause direct medical costs.^6,8^ As of 2017, acquisition costs for all DMTs were over $70 000 per year, which imposes a considerable economic burden on the US healthcare system and barriers to DMT access for PwMS.^6^

The presence and scope of unused medications in the US have come under scrutiny due to the impact on patient health outcomes, healthcare resource utilization, and environmental safety.^3,9,10^ A US survey estimated that approximately 42% of medications is wasted in households, and 67% of unused medication is disposed of or returned in its original quantity.^9^ Among all US healthcare expenditures, which total $2.3 trillion annually, more than $700 billion (≈30%) are attributable to medication waste.^9^

Medication wastage occurs among PwMS for various reasons, including reduced adherence, aggressive dispensation practices by specialty pharmacies, discontinuation of treatment shortly after initiation due to adverse events, switching DMTs before using a full prescription’s worth, and/or accumulating multiple medications as the correct therapy is identified.^3^ The quantity of unused DMTs collected from PwMS by a single provider exceeded a retail value of $6 million in 2018.^3^ A substantial proportion of MS medication wastage resulted from medication switching, and 60% of PwMS switched DMTs, more often for tolerability issues (eg, injection site reactions, hair thinning, gastrointestinal disturbances, flushing) or nonmedical reasons (eg, family planning, insurance coverage changes, or desire to switch to newly released medications) than because of inadequate disease control per clinical measures of efficacy.^3^ Globally, these data suggest that individualized treatment decisions for MS are still limited by a variety of factors including third-party administrator requirements (ie, step-therapy protocols) and gaps of knowledge involving personal characteristics, beliefs, and attitudes that may influence treatment decisions along with adherence and persistence.

Limited data exist on the financial magnitude of unused oral and self-injectable medications due to DMT switching. The objective of this exploratory study was to estimate the cost of potential medication wastage (PMW) associated with switching from an oral or self-injectable DMT among PwMS.

## METHODS

### Study Design

This retrospective cohort study by calendar year used US PharMetrics® Plus administrative commercial, Medicare Advantage, and Medicaid insurance claims data.

**Figure 1.**
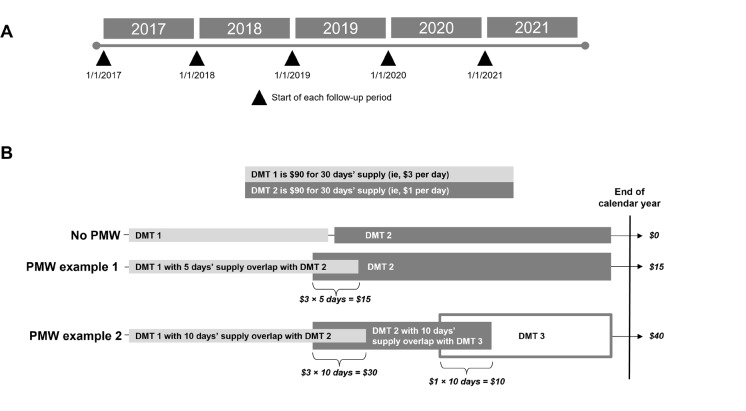
Design of Study Period (**A**) and Illustrative Examples of PMW Costs (**B**)^a^ ^a^PMW was determined only at the point of switch; the cost is based only on the prior DMT. Abbreviations: DMT, disease-modifying therapy; MS, multiple sclerosis; PMW, potential medication wastage.

The study period ranged from January 1, 2017, through December 31, 2021 (**Figure 1A**). There was no baseline period. The follow-up period was defined as 12 months of a calendar year, but PwMS could contribute data for more than 1 year.

### Study Cohort

PwMS were included if they had (1) a confirmed MS diagnosis during the follow-up year, defined either by outpatient claims at least 30 days apart or 1 inpatient claim for MS, (2) continuous enrollment for coverage for the year, and (3) use of at least 2 different DMTs during the same calendar year. PwMS were excluded if they did not use multiple DMTs within the qualifying period or if their first DMT was an IV treatment; PwMS were also censored on initiation of an IV DMT. The PMW cohort comprised individuals with an overlap in medication between switches for a given calendar year. Individuals in the non-PMW cohort did not have overlap in medication between switches for a given calendar year.

### Outcomes

The PMW and non-PMW cohorts were analyzed each year (**Figure 1B**). PMW was defined as the overlap (in days) between the prior DMT and subsequent DMT following a medication switch. Costs were derived from claims in PharMetrics® Plus. The total cost was the allowed amount, which was the contracted or accepted reimbursable amount for covered medical services or supplies that the health plan agreed to pay to service providers. The paid amount was the actual amount paid to the provider by the health plan/payer. The difference between allowed and paid was the estimated out-of-pocket cost that the patient was responsible for; this was considered an estimate because it did not capture whether or not a copay or patient assistance programs were applied. PMW cost was defined as the cost of the prior DMT during the overlap period.

Characteristics of the PMW and non-PMW cohorts, including age, sex, region, and insurance type, were evaluated. The measured outcomes were costs (in 2021 US dollars) paid by insurance and/or PwMS and switching patterns. Each of these outcomes was analyzed quantitatively. For PwMS who had 2 or more switches in any calendar year, all switches were captured in analyses of oversupply status and switching costs. For identification of the top 10 DMT switching patterns, each PwMS only contributed 1 switch per calendar year in which they were enrolled (the first switch in any calendar year was retained).

### Statistical Analysis

For the population characteristics, continuous data (age) were summarized as mean (SD) and categorical data (sex, region, payer type, and number of switches) were presented descriptively as frequency and percentage. Continuous data (ie, age) were analyzed via Wilcoxon rank sum test; categorical variables (ie, sex and region) were evaluated via Pearson’s χ^2^ test; categorical data with some sparsity (ie, payer type and number of switches) were evaluated by Fisher exact test, using R version 4.2.2.

## RESULTS

### Population Characteristics

Between 2017 and 2021, approximately 6% of PwMS in the US PharMetrics® Plus database met the inclusion criteria (ie, had claims for ≥2 DMTs in 1 year). Each calendar year, approximately 1500 PwMS were analyzed, and, ultimately, a total of 7968 PwMS were included in the analysis (**Table 1**). Approximately a quarter of PwMS with at least 2 DMTs identified were included in the PMW cohort each year: 464 of 1762 (26%) in 2017, 457 of 1947 (23%) in 2018, 419 of 1679 (25%) in 2019, 381 of 1461 (26%) in 2020, and 447 of 1782 (25%) in 2021 (**Tables 1 and S1**). The demographic characteristics between PMW and non-PMW cohorts were similar and consistent across years (**Table S1**). The mean age was 45 to 47 (SD, 11-12) years, and 70% to 78% of participants in each cohort were female (**Table S1**). PwMS with at least 2 DMTs had relatively even geographic distribution across the US, with a slight majority living in the South (29%-40%) and the fewest living in the West (10%-14%) (**Table S1**). Most PwMS (58%-66%) had commercial insurance (**Table S1**). Medicare Advantage and Medicaid insurance claims data were limited. The majority of eligible PwMS (95%) switched DMTs only once within each year (**Table S1**).

**Table 1.** Study Attrition Among People With a Confirmed MS Diagnosis^a^

	**Attrition, n (%)**				
	**2017 (N=28 372)**	**2018 (N=28 046)**	**2019 (N=28 391)**	**2020 (N=28 044)**	**2021 (N=28 307)**
≥2 different DMTs during the year	2115 (7.5)	2287 (8.2)	1956 (6.9)	1740 (6.2)	2144 (7.6)
No multiple DMTs on the same date	2105 (7.4)	2276 (8.1)	1952 (6.9)	1730 (6.2)	2133 (7.5)
No missing days’ supply	1973 (7.0)	2272 (8.1)	1949 (6.9)	1729 (6.2)	2133 (7.5)
No IV DMTs as the first DMT	1762 (6.2)	1947 (6.9)	1679 (5.9)	1461 (5.2)	1782 (6.3)

^a^Individuals could contribute data for >1 year. There were 7968 unique PwMS from 2017 to 2021. Data on DMT continuation/switching censored at the time of DMT switch to IV DMT (ie, if an individual switched to an IV DMT, oversupply/no oversupply was computed for that and prior switches, but the individual was no longer followed up after the point of this switch).

Abbreviations: DMT, disease-modifying therapy; IV, intravenous; MS, multiple sclerosis; PwMS, people with multiple sclerosis.

### Costs of PMW

The total estimated costs of PMW for insurers and estimated out-of-pocket costs for PwMS were similar across years (**Table 2**). The mean (SD) total costs of PMW were $3211 ($3015) in 2017, $3049 ($2866) in 2018, $3173 ($4040) in 2019, $3152 ($4188) in 2020, and $2964 ($4869) in 2021 (**Table 2**). The total mean (SD) costs of DMTs prior to switches were estimated to be between $8137 ($7510) in 2020 and $9339 ($5788) in 2017 (**Table 2**). The mean (SD) proportion of DMT costs attributable to PMW was consistent across years and ranged from 34% (24%) in 2021 to 38% (26%) in 2020 (**Table 2**).

**Table 2.** Total Patient-Level Costs by Year in the PMW Cohort

	**2017 (n=464)**	**2018 (n=457)**	**2019 (n=419)**	**2020 (n=381)**	**2021 (n=447)**
Total cost of PMW, mean (SD), $^a^	3211 (3015)	3049 (2866)	3173 (4040)	3152 (4188)	2964 (4869)
Total cost across DMTs prior to switch, mean (SD), $^a^	9339 (5788)	8749 (5301)	8850 (6089)	8137 (7510)	8252 (8039)
Proportion of total cost attributable to PMW, mean (SD), %	36 (24)	35 (24)	35 (25)	38 (26)	34 (24)

^a^Total costs account for both insurance and PwMS.

Abbreviations: DMT, disease-modifying therapy; PMW, potential medication wastage; PwMS, people with multiple sclerosis.

**Figure 2.**
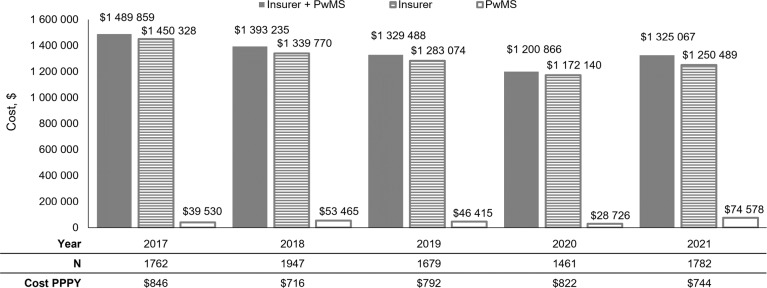
Total Overall Costs of PMW^a^ ^a^Adjusted to 2021 US dollars. Abbreviations: PMW, potential medication wastage; PPPY, per person per year; PwMS, people with multiple sclerosis.

The annual combined insurer and patient cost of PMW across all years was approximately $1.3 million (**Figure 2**). The majority of costs associated with PMW were paid by insurance ($1 172 140-$1 450 328), but PwMS still owed a substantial amount ($28 726-$74 578) (**Figure 2**). Individual costs were distributed across all PwMS with DMT switches and consistently amounted to $716 to $846 per person per year (**Figure 2**).

### DMT Switch Patterns

**Figure 3.**
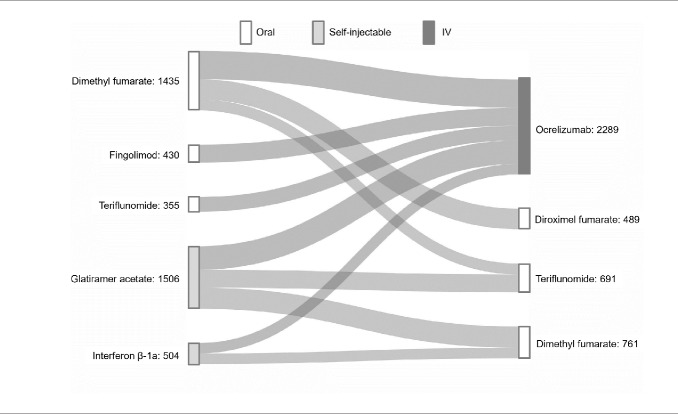
Sankey Diagram of Top 10 Switch Patterns Abbreviation: IV, intravenous.

DMT switches were captured for each individual in each calendar-year cohort (8631 total switches) and pooled to identify switch patterns (ie, initial DMT to subsequent DMT) from 2017 to 2021. The top 10 switch patterns comprised 4230 (49%) of all switches analyzed. The top 5 DMTs that PwMS switched from were dimethyl fumarate, fingolimod, teriflunomide, glatiramer acetate, and interferon β-1a (**Figure 3**). A majority of PwMS ultimately switched to ocrelizumab (54% of 4230 switches) from both oral and self-injectable DMTs (**Figure 3**). The remaining switches were to oral DMTs: diroximel fumarate, teriflunomide, and dimethyl fumarate (**Figure 3**).

## DISCUSSION

The objective of this study was to estimate PMW associated with DMT switching among PwMS with claims for at least 2 DMTs in a year. During each calendar year, patient-level costs were $8100-$9300 for DMTs from which PwMS eventually switched, including approximately $3000 incurred in PMW due to these switches. Initiation of an ideal DMT for an individual, or one that offers better adherence, would potentially result in savings for employers, insurers, and PwMS. Insurers paid most PMW-associated costs, but PwMS still faced a financial burden. PwMS have reported that out-of-pocket costs are more important to them in treatment decisions than a DMT’s efficacy or safety profile.^11^ Some PwMS will spend more than 8% of the US median household income on their medications each year, and the continually increasing DMT-related out-of-pocket costs have had measurable effects on medication adherence.^7,12–14^

Approximately half of PwMS discontinue their medication within the first 3 years of treatment^15^; this suggests that within MS care, there may be a systemic shortcoming in identification of ideal DMTs following MS diagnosis that is likely influenced by individual characteristics and circumstances.^3^ In a single-prescriber cohort study (N = 422), DMT persistence was higher for men vs women and for White PwMS vs Black or African American PwMS, and the majority of patients switched DMTs for reasons other than lack of efficacy or intolerable side effects.^3^ Alternatively, high DMT switching rates may reflect deviations between treatments recommended by specialists and insurers’ lists of preferred agents. In a retrospective cohort claims analysis (N = 12 431), 40% of PwMS did not meet adherence criteria when taking oral or self-injectable DMTs, based on prescription claims data.^16^ Prescriptions covered an average of only 265 of 365 days per year, leaving PwMS untreated for approximately 100 days per year.^16^ Adherence is a significant predictor of MS-related healthcare resource utilization and costs.^16,17^ A real-world analysis demonstrated that PwMS who demonstrated persistence and adherence to their prescribed DMT over 24 months saved an average of $20 000 on non-DMT costs.^17^

In this study, PwMS who initiated oral or self-injectable DMTs most frequently switched to ocrelizumab, which is associated with high rates of adherence. PwMS who receive injectable and oral treatments have greater risks of switching to a new DMT than those receiving infused monoclonal antibody treatments.^3^ In a real-world setting (n = 1710), PwMS who initiate ocrelizumab demonstrated 80% adherence at 24 months vs 55% with oral DMTs, 54% with other IV DMTs, and 35% with self-injectable DMTs.^18^ More stable adherence rates with ocrelizumab may be attributed to its dosing schedule. Adherence to oral or self-injectable DMTs may decrease over time^19^; Yermakov et al found 82% adherence at 6 months vs 67% at 36 months (n = 1510).^15^ IV DMTs with an infrequent dosing schedule may not be associated with decreasing adherence with long-term treatment.^20,21^

These findings should be interpreted in the context of certain limitations. First, individual PwMS were not followed up over time, and each calendar year was treated as a unit of analysis; however, over 28 000 individuals were considered over 5 years, suggesting that the trends uncovered herein were robust. We did not identify consistent associations between age, sex, or payer type and PMW; however, these and other individual characteristics (eg, race, ethnicity, or use of general vs specialty pharmacies) should be further evaluated in future work to enhance our understanding of specialty pharmacy waste resulting from DMT switches. Second, continuous eligibility for a full calendar year was required, and the study was limited to PwMS who switched from an oral or self-injectable DMT (eg, use of pills or cartridges), excluding PwMS who switched from IV DMTs because the ability to measure IV DMT waste is not feasible, which may limit the generalizability of our findings. Third, costs associated with wastage were only narrowly evaluated based on medication overlaps; to evaluate the comprehensive economic advantage of preventing DMT switching, additional benefits, including lower relapse rates, healthcare resource utilization (eg, physician visits, clinical testing, imaging), quality-of-life improvements, and employment/productivity measures, should be assessed.^16^ Notably, costs due to lost productivity comprise approximately one-third of all-cause expenditures in MS.^8^ Another limitation was that out-of-pocket costs were derived via proxy rather than directly measured. Additionally, future analyses could measure other forms of medication wastage, such as wastage that is encodable by J Codes, which were updated in 2023.^22^ Lastly, we assumed that the days’ overlap in supply was PMW, but time may have elapsed between the prescription fill and initiation of the new DMT; the individual may have used their remaining supply of the old DMT during that time. However, previous research has shown that specialty pharmacies commonly fill prescriptions for the next 3-month supply of a DMT 1 week into the current 3-month period, resulting in substantial surplus medication if a DMT switch occurs within those 6 months of supply.^3^

## CONCLUSIONS

Optimization of patient outcomes may be achieved through increased DMT adherence and reduced medication switching, in addition to reduction of costs to PwMS and third-party administrators. While most costs were paid by insurance plans, PwMS with at least 2 DMTs still owed approximately $800 associated with their DMT each year, which did not encompass additional treatment-associated costs. Dimethyl fumarate (oral), glatiramer acetate (self-injectable), and fingolimod (oral) were among the top medications that PwMS switched from and top contributors to the total cost of PMW. IV DMTs may contribute to PMW less than oral or self-injectable DMTs. Additional studies are needed to further validate these findings, explore relationships between medication switching and personal characteristics, and advocate for policy changes supporting use of first-line DMTs that are most appropriate for PwMS.

### Conflict-of-Interest Disclosures

N.G.B., A.P., R.S., and I.A. are employees of Genentech, Inc., and shareholders in F. Hoffmann-La Roche Ltd. D.T.O. received personal compensation for consulting and advisory services from Eisai, EMD Serono, Genentech, Inc., Genzyme/Sanofi, Immunic Therapeutics, Moderna, and RVL Pharmaceuticals, Inc., and research support from Biogen, EMD Serono/Merck, and Novartis. D.T.O. has issued national and international patents along with pending patents related to the enclosed work as well as other developed technologies. D.T.O. received royalties for intellectual property licensed by The Board of Regents of The University of Texas System. D.T.O. is the founder of Revert Health Inc.

### Author Contributions

N.G.B., A.P., R.S., and D.T.O. conceived and designed the study, collected data and performed analysis. All authors reviewed and approved each draft of the manuscript.

### Data Sharing Statement

The IQVIA PharMetrics® Plus database analyzed during this study is not publicly available as this is proprietary information.

## Supplementary Material

Online Supplementary Material
