# Supplementary material for: Costs of Potential Medication Wastage Due to Switching Treatment Among People With Multiple Sclerosis
Source: J Health Econ Outcomes Res. 2024 Oct 28;11(2):103–8. doi: 10.36469/001c.123336 (PMC11523564; doi:10.36469/001c.123336)
Supplement: Online Supplementary Material [file jheor_2024_11_2_123336_251073.pdf]

Journal of Health Economics and Outcomes Research

Online Supplementary Material

Costs of Potential Medication Wastage Due to Switching Treatment Among People With Multiple Sclerosis. *JHEOR*. 2024;11(2):103-108. doi:10.36469/jheor.2024.123336

Table S1: Demographic Characteristics Between PMW and Non-PMW Cohorts

This supplementary material has been provided by the authors to give readers additional information about their work.

This is an open-access article distributed under the terms of the Creative Commons Attribution 4.0 International License (CCBY-4.0). View this license's legal deed at http://creativecommons.org/licenses/by/4.0 and legal code at http://creativecommons.org/licenses/by/4.0/legalcode for more information.

Table S1. Demographic Characteristics Between PMW and Non-PMW Cohorts

**Characteristic**	**2017 (N=1762)**			**2018 (N=1947)**			**2019 (N=1679)**			**2020 (N=1461)**			**2021 (N=1782)**		
	**No PMW**	**PMW**	***P* Value**[a]	**No PMW**	**PMW**	***P* Value**[a]	**No PMW**	**PMW**	***P* Value**[a]	**No PMW**	**PMW**	***P* Value**[a]	**No PMW**	**PMW**	***P* Value**[a]
n (%)	1298 (74)	464 (26)	–	1490 (77)	457 (23)	–	1260 (75)	419 (25)	–	1080 (74)	381 (26)	1335 (75)	447 (25)	–	
Age, mean (SD), years	46 (12)	46 (11)	0.8	46 (11)	47 (11)	.5	45 (11)	46 (11)	.09	46 (11)	47 (11)	0.4	47 (11)	47 (11)	.5
Female, n (%)	1003 (77)	362 (78)	0.7	1135 (76)	318 (70)	.005	963 (76)	318 (76)	.8	817 (76)	294 (77)	0.6	1023 (77)	350 (78)	.5
Region, n (%)															
East	338 (26)	129 (28)		359 (24)	102 (22)		235 (19)	98 (23)		218 (20)	65 (17)		276 (21)	97 (22)	
Midwest	356 (27)	123 (27)	0.9	447 (30)	125 (27)	.4	389 (31)	100 (24)	.008	327 (30)	130 (34)	0.4	459 (34)	161 (36)	.08
South	458 (35)	158 (34)		527 (35)	182 (40)		493 (39)	159 (38)		426 (40)	145 (38)		458 (34)	128 (29)	
West	145 (11)	52 (11)		157 (11)	48 (11)		142 (11)	61 (15)		108 (10)	41 (11)		140 (11)	61 (14)	
Payer type, n (%)															
Commercial	804 (62)	295 (64)		959 (64)	293 (64)		788 (63)	275 (66)		690 (64)	236 (62)		855 (64)	261 (58)	
Medicaid	15 (1)	8 (2)		15 (1)	2 (<1)		3 (<1)	1 (<1)		2 (<1)	2 (1)		6 (<1)	6 (1)	
Medicare Advantage	40 (3)	18 (4)	0.4	53 (4)	9 (2)	.2	36 (3)	17 (4)	.2	44 (4)	13 (3)	.3	41 (3)	33 (7)	<.001
Medicare Supplemental	13 (1)	1 (<1)		11 (<1)	7 (2)		2 (<1)	2 (<1)		1 (<1)	2 (1)		6 (<1)	2 (<1)	
Self-insurance	420 (32)	140 (30)		445 (30)	144 (32)		431 (34)	124 (30)		343 (32)	128 (34)		425 (32)	145 (32)	
Switches, n (%)															
1	1205 (93)	437 (94)		1401 (94)	435 (95)		1195 (95)	404 (96)		1021 (95)	360 (95)		1262 (95)	423 (95)	
2	90 (7)	17 (4)	NE[b]	88 (6)	16 (4)	NE[b]	63 (5)	11 (3)	NE[b]	55 (5)	16 (4)	NE[b]	69 (5)	15 (3)	NE[b]
≥3	3 (<1)	10 (2)	NE[b]	1 (<1)	6 (1)	NE[b]	2 (<1)	4 (1)	NE[b]	4 (<1)	5 (1)	NE[b]	4 (<1)	9 (2)	NE[b]

[a]*P* values were determined by Wilcoxon rank sum test, Pearson χ^2 test, or Fisher exact test and compared the no PMW and PMW cohorts within each year.
[b]Not evaluated due to small numbers in the 2 and ≥3 switch categories.
Abbreviations: NE, not evaluated; PMW, potential medication wastage; PwMS, people with multiple sclerosis.
